# Role of Dsg1- and Dsg3-Mediated Signaling in Pemphigus Autoantibody-Induced Loss of Keratinocyte Cohesion

**DOI:** 10.3389/fimmu.2019.01128

**Published:** 2019-05-24

**Authors:** Elias Walter, Franziska Vielmuth, Marie-Therès Wanuske, Matthias Seifert, Robert Pollmann, Rüdiger Eming, Jens Waschke

**Affiliations:** ^1^Institute of Anatomy and Cell Biology, Ludwig-Maximilians-Universität München, Munich, Germany; ^2^Department of Dermatology and Allergology, Philipps-Universität Marburg, Marburg, Germany

**Keywords:** autoimmunity, pemphigus, keratinocytes, desmoglein, signaling, CRISPR/Cas9

## Abstract

Pemphigus is an autoimmune dermatosis in which mucocutaneous blisters are induced primarily by autoantibodies against Desmoglein (Dsg) 1 and 3. Pemphigus vulgaris (PV) usually is associated with autoantibodies against Dsg3 whereas pemphigus foliaceus (PF) patients present autoantibodies against Dsg1. Several signaling pathways were proposed to cause loss of keratinocyte adhesion. However, relevance of different signaling pathways and role of Dsg1 and 3 to trigger signaling are not fully understood. Here, we show that Ca^2+^ chelation reduced PV-IgG- and PF-IgG-mediated loss of HaCaT keratinocyte cohesion whereas EGFR inhibition did not inhibit effects of PF-IgG. PV-IgG activated EGFR in a Src-dependent manner whereas both PV-IgG and PF-IgG caused Ca^2+^ influx independent of EGFR. ERK activation was Src-dependent in response to PV-IgG but not PF-IgG. To delineate the roles of Dsg isoforms to trigger signaling pathways, Dsg3- and Dsg2-deficient HaCaT keratinocyte cell lines were generated using CRISPR/Cas9. Dsg3- but not Dsg2-deficient cells were protected against PV-IgG-induced loss of cell adhesion. Ca^2+^ influx and ERK activation in response to PF-IgG were preserved in both cell lines.

## 1. Introduction

Pemphigus is an autoimmune blistering disease affecting mucous membranes and the epidermis (1). The pathogenesis of pemphigus is complex and includes genetic factors facilitating production of autoantibodies and enhancing susceptibility of keratinocytes for loss of cell adhesion (2, 3). Factors contributing to blister formation include antibodies against the desmosomal cadherins desmoglein (Dsg) 1 and 3 (1) as well as non-antibody factors such as soluble Fas ligand (4). Autoantibodies against other keratinocyte antigens are also found in patients' sera and blister fluid and may augment pathogenic effects, however, their role for blister formation is undefined at present (5, 6). Thus, it is generally accepted that antibodies against desmosomal cadherins are sufficient to cause acantholysis in pemphigus (7). Desmosomes are highly specialized adhesive contacts most abundant in tissues subjected to high mechanical stress such as the epidermis and heart muscle (8). Indeed, it was demonstrated recently that desmosomes bear mechanical load primarily when cells are exposed to external mechanical stress (9). Usually, in pemphigus vulgaris a mucosal-dominant phenotype (m-PV) is paralleled by autoantibodies against Dsg3 whereas epidermal involvement in PV (mc-PV) is in addition associated with formation of autoantibodies against Dsg1 which can also be found in pemphigus foliaceus (PF) (10). The mechanisms by which antibodies against desmosomal cadherins induce loss of cell adhesion are multiple and comprise direct inhibition of Dsg3 binding as well as a large number of signaling pathways which finally interfere with desmosome turn-over (7, 11, 12). In this context, desmosomal cadherins have been proposed to serve as signaling hubs to coordinate cell adhesion with cell-specific functions such as wound-healing in keratinocytes (13, 14). However, despite the intriguing correlation between clinical phenotype and autoantibody profiles, the factors defining the different clinical phenotypes in pemphigus are poorly understood. Previously, it was reported that autoantibody profiles also correlate with different subsets of signaling pathways engaged suggesting that signaling pattern define the clinical course of the disease (15). Antibodies specific for Dsg3 such as the mouse pemphigus antibody AK23 (16) similar to m-PV-IgG was sufficient to activate p38MAPK and Src whereas Ca^2+^ influx and ERK activation were detectable only when antibodies against Dsg1 were present in patients IgG fractions such as mc-PV-IgG and PF-IgG (15). Here, we further characterized the role of the different signaling mechanisms and of antibodies against Dsg1 and Dsg3. We observed that signaling mechanisms such as EGFR and ERK activation or influx of Ca^2+^ not only correlated with autoantibody profiles but also were at least in part dependent on other signaling molecules such as Src. We established human keratinocyte cell lines deficient for Dsg3 and observed that in the absence of Dsg3 PV-IgG-induced loss of cell adhesion was abolished indicating that autoantibodies against Dsg3 are required for this process.

## 2. Materials and Methods

### 2.1. Cell Culture

HaCaT (Human adult high Calcium low Temperature) keratinocytes (17) were cultured in Dulbecco's Modified Eagle Medium (DMEM) containing 10% FCS (Biochrom, Berlin, Germany), 50 U/ml penicillin and 50 mg/ml streptomycin (AppliChem, Darmstadt, Germany) in a humidified atmosphere of 5% CO_2_ and 37°C. On the day cells reached confluency medium was changed and experiments were performed 24 h later.

### 2.2. Pemphigus Sera and IgG Purification

Patient sera were acquired from the Dermatology department of the Philipps Universität Marburg. Patients and donors gave written consent for research use. A positive vote of the Ethics Committee from the Medical Faculty of the University of Marburg was given. During disease diagnosis the phenotype was histologically and immunologically confirmed (Table 1). All patients presented phenotype as well as antibody profile typical clinical features. Sera of patients and healthy volunteers were purified to IgG-fractions for experimental use by protein A affinity chromatography as described previously (18). All IgG fractions including c-IgG were subjected to ELISA assays (Euroimmun, Lübeck, Germany) to determine Dsg3 and Dsg1 antibody profiles. All scores below a 20 U/ml cut-off were considered as negative.

**Table 1:** Antibody profiles of IgG-fractions determined by Dsg1 and Dsg3 ELISA and their clinical phenotypes.

	**ELISA score (U/ml)**		**Clinical phenotype**
	**aDsg1**	**aDsg3**	
c-IgG	–	–	healthy control
m-PV	–	162.93	mucosal PV
mc-PV	153.76	172.53	mucocutaneous PV
mc-PV2	101.18	106.72	mucocutaneous PV
PF	215.34	–	cutaneous PF

*Cut off values are considered as 20 U/ml*.

### 2.3. Pharmacological Mediators

Signaling pathway inhibition was accomplished using pharmacological mediators which were used at the indicated concentrations and time periods prior to treatment to assure sufficient effects (Table 2). Vehicle control conditions were treated with the respective DMSO concentration.

**Table 2:** Pharmacological mediators with their target molecule, concentration, pretreatment as well as commercial origin.

**Inhibitor**	**Target**	**Concentration (μM)**	**Pre-treatment (h)**	**Origin**
U0126	MEK 1/2	5	1	New England Biolabs, Ipswich, USA
PP2	Src-family kinases	10	1	Merck Millipore, Billerica, USA
BAPTA-AM	Intracellular Ca^2+^ chelation	200	4	Merck Millipore, Billerica, USA
Erlotinib	EGFR	2.5	1	Santa Cruz Biotechnology,Santa Cruz, USA

### 2.4. Immunostaining

Cell monolayers were grown on glass coverslips until confluency and fixed with 2% formaldehyde from paraformaldehyde for 10 min. After permeabilization of cells with 0.1% Triton X-100 in PBS for 5 min, blocking was achieved by incubation of 3% bovine serum albumin (BSA) and 1% normal goat serum (NGS) in PBS for 60 min. The following primary antibodies were incubated overnight at 4°C: anti-Dsg3 mAb (clone 5G11, Invitrogen, Carlsbad, CA, USA), anti-Dsg2 mAb (OriGene, Herford, Germany), anti-E-Cadherin mAb (BD Transduction Laboratories, Heidelberg, Germany), anti-PG mAb (Progen, Heidelberg, Germany), anti-DP mAb (NW6, kind gift from Kathleen J. Green, Northwestern University, Chicago, USA). Cy3 coupled goat anti-rabbit or goat anti-mouse secondary antibodies (Dianova, Hamburg, Germany) were used to visualize the respective protein by incubation for 1 h at room temperature. Finally, cover slips were mounted with 2% n-propyl-gallate (NPG) on glass coverslides and evaluated on a SP5.II confocal microscope equipped with a 63x NA 1.4 PL APO objective (Leica, Mannheim, Germany).

### 2.5. Dispase-Based Dissociation Assay

Confluent monolayers were subjected to treatment as indicated in the respective experiment. Afterwards, cells were detached from the well bottom by application of 200 μl Dispase-II solution (Sigma-Aldrich) for 20 min. Following successful detachment, 350 μl Hank's Balanced Salt Solution (HBSS) was used to substitute the enzyme. Defined mechanical shear stress was applied with an electrical pipette (Finnpipette Novus, ThermoFisher, Waltham, USA). Cell-sheet fragments correlate with loss of adhesion (19) and were counted under a binocular microscope (Stemi 508, Zeiss, Jena, Germany). Pictures were taken with a Canon EOS 600D camera (Krefeld, Germany).

### 2.6. Cell Lysis, Gel Electrophoresis, and Western Blotting

After reaching confluency, cell monolayers were switched to 300 μl fresh medium for 24 h. IgG fractions and mediators were incubated as indicated. The following protocol was performed as described previously (15). In short, cell lysates were separated into a soluble cytosolic and insoluble cytoskeletal bound fraction by incubation of triton extraction buffer (0.5% Triton X-100, 50 mmol/l MES, 25 mmol/l EGTA, 5 mmol/l MgCl_2_, pH 6.8, 0.1% of each Pepstatin, Aprotinin and Leupeptin, 1% PMSF) for 15 min on ice under gentle shaking. Cells were harvested with a cell scraper and the insoluble pellet was sedimented by centrifugation for 10 min at 4°C as well as 14,000 rcf. Supernatants were collected and the pellet was resuspended in SDS lysis buffer (25 mM Hepes, 2 mM EDTA, 25 mM NaF, 1% SDS, pH 7.6, complete Protease Inhibitors). Protein amount was determined with a commercial Pierce BCA Protein Assay Kit and lysates were subjected to Western blot analysis with a standard wet blotting protocol on nitrocellulose membranes (Life Technologies, Carlsbad, USA). Membranes were blocked by 5% bovine serum albumin (BSA) in Tris-buffered saline with 0.05% Tween (TBS-T) for 1 h at room temperature and following primary antibodies were used overnight at 4°C in blocking solution: phospho-EGF Receptor mAb (Tyr845) (Cell Signaling Technologies, Danvers, USA), EGF Receptor mAb (CST), phospho-p44/42 MAPK mAb (CST), p44/42 MAPK mAb (CST), GAPDH (Santa Cruz, Heidelberg, Germany), Desmoplakin I/II (H-300) (Santa Cruz), α-Tubulin (Abcam, Cambridge, UK), Dsg3 pAb (Biozol, Eching, Germany), PG (Progen, Heidelberg, Germany), E-Cad (BD Transduction), Dsg2 (OriGene, Herford, Germany), Desmocollin (Dsc) 3 (Progen). Horseradish-peroxidase coupled secondary antibodies (Dianova, Hamburg, Germany) from the respective species were used in TBS-T for 1 h at room temperature and visualized by a self-made ECL solution on a FluorchemE (Protein Simple, San Jose, USA) developer. Every analyzed signaling protein was exclusively localized in the soluble fraction. Therefore, only this fraction is shown in most figures.

### 2.7. Ratiometric Intracellular Ca^2+^ Measurements

Fura-2AM (ThermoFisher) was used to determine the intracellular Ca^2+^ concentration in real time. Cells were therefore grown in an 8-Well μ-slide (Ibidi, Martinsried, Germany) until confluency. Mediators were used before the Fura-2AM dye was applied and were also present during dye loading. A mix of 1 μM Fura-2AM and 0.02% Pluronic (ThermoFisher) was applied for 20 min in measurement buffer (140 mM NaCl, 3.6 mM KCl, 2.6 mM CaCl_2_(H_2_O)_2_, 0.5 mM NaH_2_PO_4_(H_2_O)_2_, 2 mM NaHCO_3_, HEPES and 5 mM D+ Glucose) at 37°C to facilitate transfer of the dye into cells. After replacing the solution with fresh measurement buffer cells were measured with MetaFluor (Moleculardevices, San Jóse, USA) on an Axio Observer A1 (Zeiss, Jena, Germany) with a Polychrome V (Till Photonics, Planegg, Germany), a CoolSNAP-Hq2 digital camera (Photometrics, Tucson, USA) and a Fura-2 filter set.

### 2.8. Atomic Force Microscopy

AFM data were acquired using a NanoWizard^Ⓡ^ 3 AFM (JPK-Instruments, Berlin, Germany) mounted on an inverted optical microscope (Carl Zeiss, Jena, Germany), which allowed the selection of the scanning area by visualizing the cells with a 63x objective. The pyramidal-shaped D-Tip of Si_3_N_4_ MLCT cantilevers (Bruker, Mannheim, Germany) with a nominal spring constant of 0.03 N/m and tip radius of 20 nm were used for all experiments. Cantilevers were functionalized with recombinant Dsg3-Fc (at a concentration of 0.15mg/ml) using a flexible heterobifunctional acetal-polyethylenglycol (PEG) linker (Gruber Lab, Institute of Biophysics, Linz, Austria) following a well-established protocol (20). Force-distance curves were sampled in force-mapping mode in which a picture is composed of single pixels, each containing the information of one force-distance curve. To measure Dsg3 single molecule interactions HaCaT cells were measured in DMEM containing 1.8 mM Ca^2+^ at 37°C after incubation with the respective conditions. As pathogenic aDsg3 Abs directly interfere with Dsg3 interactions (21, 22) AFM cantilevers were removed during Ab incubation and residual antibodies in the medium were vigorously cleared before reintroducing the AFM tip to avoid antibody binding to the scanning tip. Force maps were acquired at small areas along cell borders (5 × 2.5 μm) at a resolution of 50 × 25 pixels each. The setpoint was set to 0.2 nN to avoid damage of the cells during measurements. Further, a Z-length of 1.5 μm, a pulling speed of 10 μm/s and a resting contact time of 0.1 s were applied throughout all measurements.

### 2.9. CRISPR/Cas9 Mediated Gene Editing

Dsg3 and Dsg2 HaCaT knock out cell lines were generated utilizing the CRISPR/Cas9 mediated gene editing system. HaCaT keratinocytes were therefore purchased freshly and used at passage 32. Two expression vectors encoding *Streptococcus pyogenes* Cas9 coupled to green fluorescent protein (GFP) (pCMV-Cas9-GFP) with different target sites for each protein of interest were purchased (Sigma-Aldrich, St. Louis, USA) and chosen to specifically induce a double strand break at the beginning of the protein resulting in non-homologous end joining (NHEJ) repairs as indicated in Figure 4 (Target ID: Dsg2: HS0000249131, HS0000249134; Dsg3: HS0000249170, HS0000249174). The plasmid was transiently introduced into cells using Lipofectamin-2000 in Opti-MEM as instructed by the manual (ThermoFisher). Sub cloning was initiated after an expression period of 24 h by sorting single GFP-positive cells into five 96-well plates by a FACSAria III (BD Transduction) cell sorting unit for each transfection. The medium was renewed every third day for a time span of 4 weeks and wells were inspected for monoclonal cultures every week followed by individual expansion to a bigger culture dish on demand. Eventually, around 40 different monoclonal clones for each target site could be evaluated for the absence of either Dsg3 or Dsg2 by immunoblot as well as immunostaining. Afterwards, genomic DNA was extracted using a standard Phenol-Chloroform DNA extraction protocol and send for Sanger sequencing with an area of 500 base pairs flanking both ends of the target site (Eurofins, Ebersberg, Germany). Results were aligned to the known DNA sequence and alleles separated by hand in case of heterozygous mutations.

### 2.10. Analysis and Statistics

Images and figures were processed using Photoshop CC (Adobe Creative Cloud, Adobe, San Jóse, USA). The blot analysis function in ImageJ (Wayne Rasband, https://imagej.nih.gov/ij) was used to quantify protein density in immunoblots and graphs were generated in Graphpad Prism (GraphPad Software, San Diego, USA). Each n represents an independent experiment. Statistical Analysis was performed in Prism using either paired one-way ANOVA corrected by Dunett's test for multiple comparisons or paired two-way ANOVA corrected by Fisher's LSD test for experiments with separate factors as indicated in the figure legends. Statistical significance was assumed at *p* ≤ 0.05. Bar diagrams are presented as mean ± standard error.

## 3. Results

### 3.1. Relevance of Ca^2+^ and EGFR Signaling for Pemphigus Autoantibody-Induced Loss of Cell Adhesion

**Figure 1:**
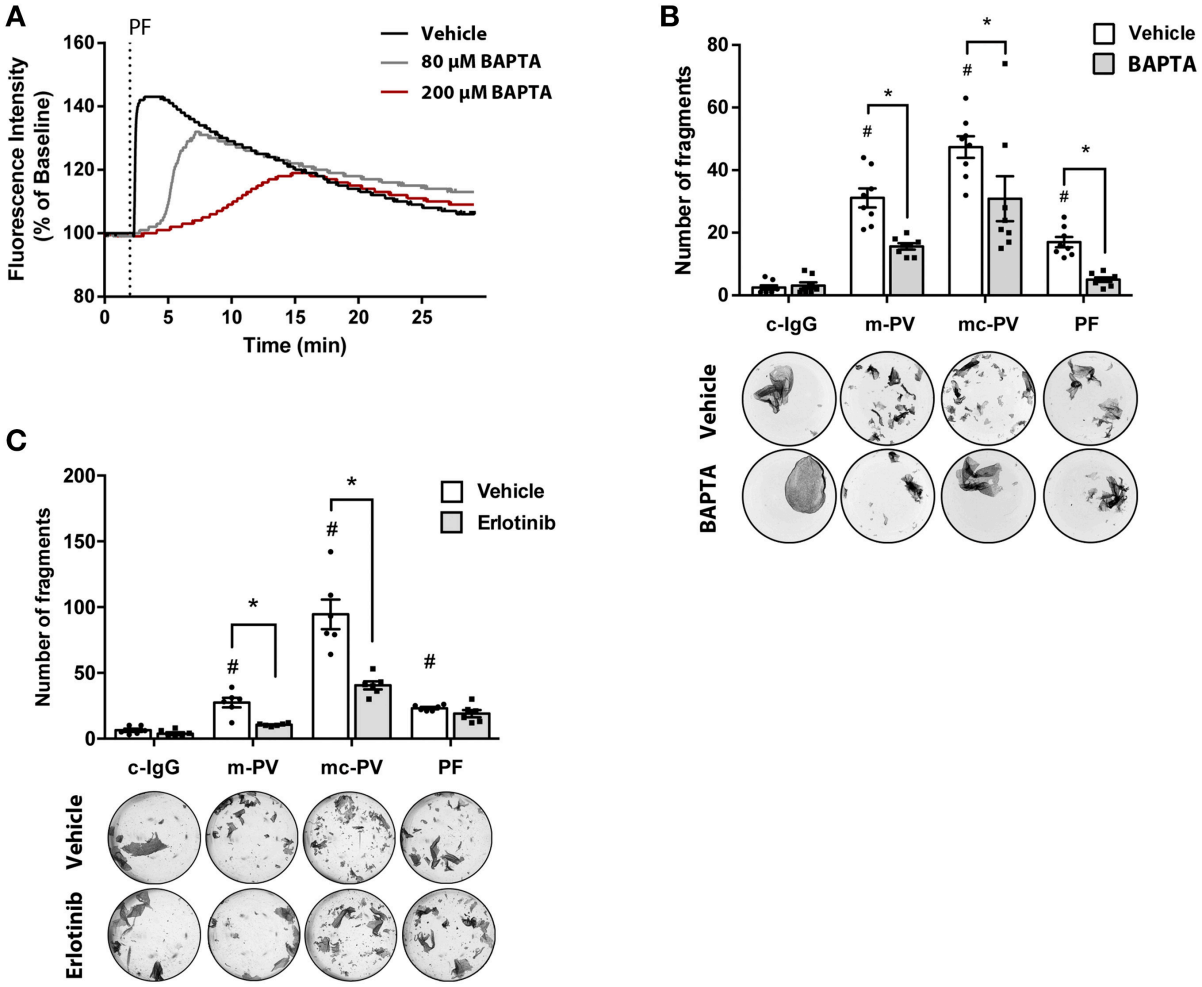
Relevance of EGFR and Ca^2+^ signaling for pemphigus autoantibody-induced loss of cell adhesion. **(A)** Fura measurements of HaCaT cells pretreated with 80 μM as well as 200 μM BAPTA-AM for 4 h to determine Ca^2+^ chelation efficiency after PF-IgG treatment (*n* = 4, representative graph). HaCaT keratinocytes were subjected to dispase assays following incubation with different IgG fractions in presence or absence of pharmacological inhibitors to evaluate the relevance of Ca^2+^ influx as well as the EGFR signaling pathway for loss of keratinocyte adhesion. **(B)** Fragment numbers revealed reduced loss of adhesion under all conditions after chelation of intracellular Ca^2+^ by BAPTA (*n* = 8, two-way ANOVA, #*p* ≤ 0.05 vs. vehicle c-IgG, *p ≤ 0.05) **(C)** Inhibition of EGFR by Erlotinib for 1 h followed by addition of IgG fractions for 2 h revealed a decrease of fragments in monolayers treated with m-PV-IgG as well as mc-PV-IgG but not PF-IgG (*n* = 6, two-way ANOVA, #*p* ≤ 0.05 vs. vehicle c-IgG, *p ≤ 0.05).

The relevance of signaling pathways during the pathogenesis of pemphigus is widely accepted (7). Recently, we reported pemphigus phenotype-specific differences in the roles of signaling pathways for loss of adhesion in HaCaT as well as primary normal human epidermal keratinocytes (NHEK) (15). In this study, we observed that Ca^2+^ influx was associated with autoantibodies against Dsg1 in patients' IgG. Others have reported that epidermal growth factor receptor (EGFR) is activated by AK23, a murine pathogenic Dsg3-specific antibody (16, 23). Therefore, we investigated the relevance of Ca^2+^ influx and EGFR signaling for loss of keratinocyte adhesion in response to IgG fractions containing different profiles of aDsg1 and aDsg3 antibodies from patients suffering from m-PV, mc-PV and PF in dispase-based dissociation assays. First, Fura measurements were performed to evaluate the efficiency of BAPTA-AM. Therefore, HaCaT keratinocytes were treated for 4 h with BAPTA-AM at different concentrations. A concentration of 200 μM BAPTA-AM was suited best to block PF-IgG induced Ca^2+^-influx (Figure 1A). In Dispase assays this concentration was effective to reduce loss of cell cohesion by approximately 40% in all conditions compared to conditions incubated with autoantibodies alone (Figure 1B). Inhibition of EGFR using Erlotinib, a specific EGFR tyrosine kinase inhibitor, reduced loss of adhesion only in response to IgG fractions containing autoantibodies targeting Dsg3, specifically in response to m-PV-IgG and mc-PV-IgG but not to PF-IgG (Figure 1C).

### 3.2. Time Dependency of Pemphigus-IgG Mediated EGFR and ERK Activation

**Figure 2:**
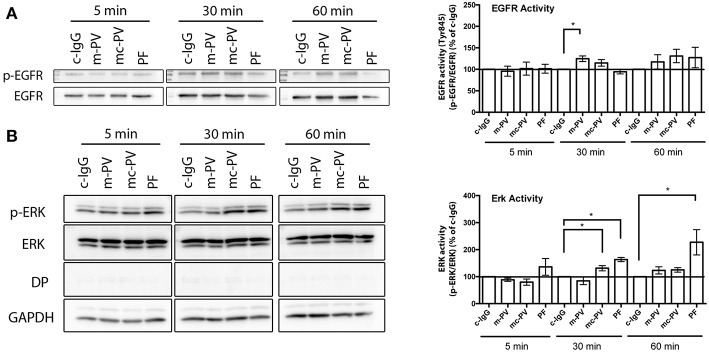
Time course of pemphigus IgG-mediated EGFR and ERK activation. Confluent HaCaT cell monolayers were incubated with different IgG fractions for 5 min, 30 min as well as 60 min to evaluate activation due to phosphorylation of **(A)** EGFR and **(B)** ERK in immunoblots. EGFR was activated after 30 min by IgG fractions containing antibodies against Dsg3 (*n* ≥ 7, *p ≤ 0.05, one-way ANOVA, normalized to respective c-IgG). ERK was activated only when antibodies targeting Dsg1 were present (*n* ≥ 7, *p ≤ 0.05, one-way ANOVA, normalized to respective c-IgG).

**Figure 3:**
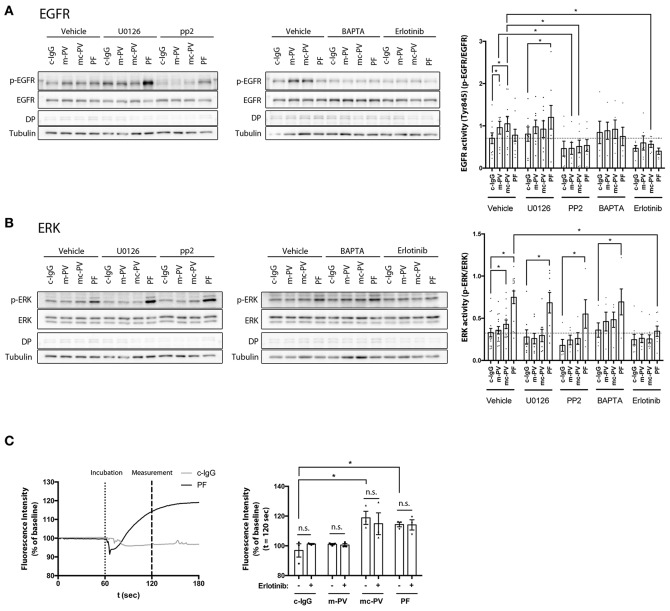
Interdependency of signaling pathways activated by pemphigus autoantibodies. Immunoblots of cell monolayers pretreated with either pharmacological inhibitors for 1 h or BAPTA-AM for 4 h followed by IgG fractions for 30 min. **(A)** EGFR activity was reduced after Src inhibition by PP2 (*n* ≥ 6, *p ≤ 0.05, two-way ANOVA) **(B)** After inhibition of EGFR by Erlotinib, ERK phosphorylation was significantly reduced in cells treated with PF-IgG. (*n* ≥ 6, p ≤ 0.05, two-way ANOVA) **(C)** Ca^2+^ influx was measured by Fura-2 ratiometric fluorescence imaging and found to be independent of EGFR (*n* = 3, *p ≤ 0.05, two-way ANOVA) Representative graph with experimental setup on the left and analysis at *t* = 120 s on the right. Dotted line indicates application of the respective IgG fraction.

Ca^2+^ influx is known to occur within 60 s after application of antibodies (24, 25), which is also supported by data from this study (Figure 3C). Next, we determined the kinetics of autoantibody-induced activation of EGFR in comparison to ERK, which we previously observed to correlate with autoantibodies against Dsg1 (15). HaCaT cells were incubated with the different IgG fractions for 5 min, 30 min as well as for 60 min. Afterwards, cell lysates were subjected to Triton fractionation and Western blotting to determine the phosphorylation of EGFR at the Src-dependent site Y845 as well as of ERK. Of note, a marked EGFR activation was detectable after 30 and 60 min but only showed a significant increase when cells were incubated with m-PV-IgG for 30 min in the soluble pool (Figure 2A). In contrast, ERK activation in response to PF-IgG was observed at all time-points but significant only after 30 and 60 min also in the soluble fraction (Figure 2B). Mc-PV-IgG-induced activation of ERK was significant after 30 min. From these experiments we concluded that for the following experiments studying the interdependence of signaling pathways, 30 min would be most suitable.

### 3.3. Interdependency of Signaling Pathways Involved in Pemphigus Autoantibody-Induced Loss of Cell Adhesion

We tested the dependency of EGFR and ERK activation on each other as well as on Src and Ca^2+^ influx after 30 min of autoantibody incubation (Figures 3A,B). In addition to Erlotinib and BAPTA-AM, PP2, a Src family kinase inhibitor, and U0126, an inhibitor of MEK upstream of ERK, were applied in parallel to the different IgG fractions. In absence of inhibitors and BAPTA-AM, m-PV-IgG and mc-PV significantly enhanced EGFR phosphorylation (Figure 3A). Activation was abolished when PP2 or Erlotinib were added. Chelation of Ca^2+^ enhanced the variability of EGFR phosphorylation, which was not significantly different to experiments using control IgG. Interestingly, after inhibition of MEK using U0126, PF-IgG-induced EGFR phosphorylation was increased whereas PV-IgG-induced activation was not. ERK activation was significant following incubation with mc-PV-IgG or PF-IgG but not m-PV-IgG (Figure 3B). Moreover, Erlotinib, PP2 and U0126 prevented mc-PV-IgG-induced ERK activation whereas ERK phosphorylation in response to PF-IgG was blunted by Erlotinib only. After chelation of Ca^2+^, PV-IgG-induced ERK activation was variable but not significant, whereas PF-IgG-induced ERK activation was significant. Ca^2+^ influx has been recognized down-stream of EGFR activation (26). Therefore, we employed Fura-2 ratiometric measurements after application of autoantibodies in presence or absence of Erlotinib. A baseline was established for 60 s in HaCaT keratinocytes, followed by application of either c-IgG, m-PV, mc-PV or PF for another 120 s. Intracellular Ca^2+^ concentrations were evaluated 60 s after addition of IgG fractions. Both, mc-PV-IgG and PF-IgG but not m-PV-IgG were effective to induce a transient Ca^2+^ influx which was not affected by inhibition of EGFR by Erlotinib (Figure 3C). Taken together, these results indicate that PV-IgG-induced activation of both EGFR and ERK are Src- and EGFR kinase-dependent whereas PF-IgG-induced ERK activation is completely independent of Src, MEK and Ca^2+^. Moreover, mc-PV-IgG- and PF-IgG-induced Ca^2+^ influx appears not to be related to EGFR signaling.

### 3.4. EGFR Activation Reduces Binding Frequency but Not Binding Strength of Dsg3 Interactions on Living HaCaT Keratinocytes

**Figure 4:**
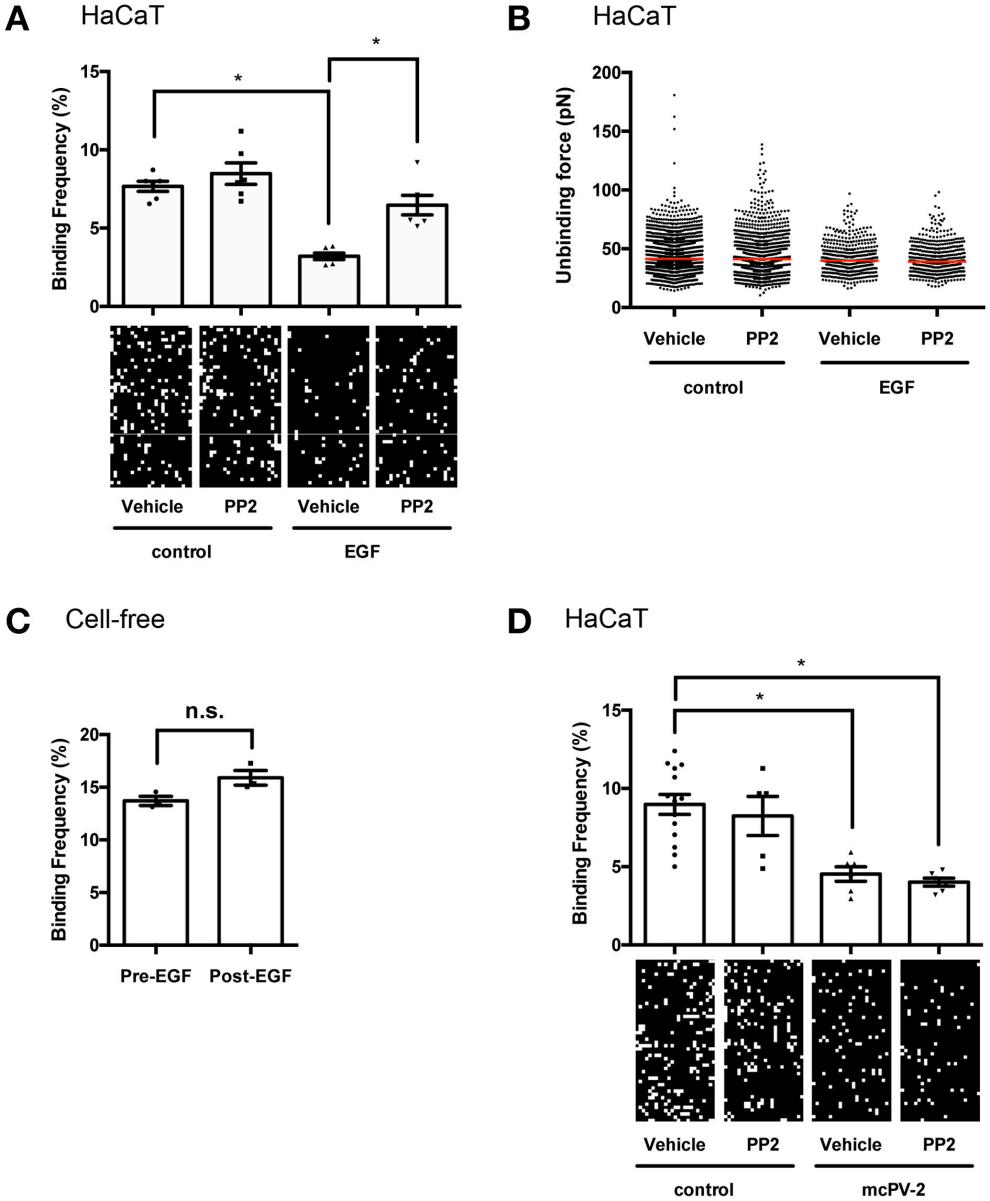
EGFR activation reduces binding frequency of Dsg3 interactions on living HaCaT keratinocytes. **(A)** Atomic force microscopy (AFM) adhesion measurements on cell borders of living HaCaT keratinocytes using a Dsg3 Fc-functionalized tip and 1 h incubation of EGF with representative force maps. A reduction in binding frequency is observable in a Src-dependent manner, (*n* = 3 with two separate cell borders per experiment, one-way ANOVA, *p ≤ 0.05) whereas **(B)** binding forces remained unaffected. **(C)** Cell-free AFM measurements on Dsg3 Fc-functionalized mica sheets prove that reduction in binding frequency is not induced by direct inhibition (*n* = 3, *t*-test, *p ≤ 0.05) **(D)** Binding frequency was reduced in HaCaT cells treated for 1 h with mc-PV2-IgG independently of Src (*n* ≥ 3, with two separate cell borders per experiment, one-way ANOVA, *p ≤ 0.05).

The mechanism by which EGFR activation reduces desmosome adhesion in pemphigus is not fully understood. It was shown previously that stimulation of EGFR by EGF reduced Dsg2 binding frequency on DLD1 enterocytes (27). Thus, we hypothesized that Dsg3 in keratinocytes may also be regulated by EGFR and evaluated Dsg3 single molecule binding properties at cell borders of living HaCaT cells before and after stimulation with EGF by atomic force microscopy (AFM). EGFR activation reduced Dsg3 binding frequency on keratinocytes significantly from 7.67 to 3.20% in a Src-dependent manner because inhibition by PP2 restored Dsg3 binding frequency to 8.48% (Figure 4A). The unbinding force remained unchanged at a median of around 40 pN (Figure 4B). To prove that the reduction in binding frequency is mediated by cellular processes and direct inhibition of Dsg3 by EGF, cell-free measurements using mica sheets functionalized with Dsg3-Fc were performed. Homophilic Dsg3 interactions were determined before as well as after EGF incubation for 1 h yielding similar results indicating that cellular mechanisms are required for reduction of Dsg3 binding (Figure 4C). Lastly, PP2 was used together with mc-PV2-IgG to evaluate if Src inhibition modulates PV-IgG-mediated direct inhibition of Dsg3 interactions. Indeed, mc-PV2-IgG reduced Dsg3 binding from 8.89 to 4.53% which in line with previous studies and most likely caused by antibody-induced steric hindrance (28, 29). However, loss of Dsg3 binding was not affected by PP2 (Figure 4D). Taken together, these results demonstrate that, on the molecular level, EGFR via Src regulates Dsg3 adhesion but inhibition of this mechanism does not interfere with autoantibody-induced direct inhibition.

### 3.5. Role of Dsg3 for Pemphigus Autoantibody-Induced Loss of Keratinocyte Adhesion

**Figure 5:**
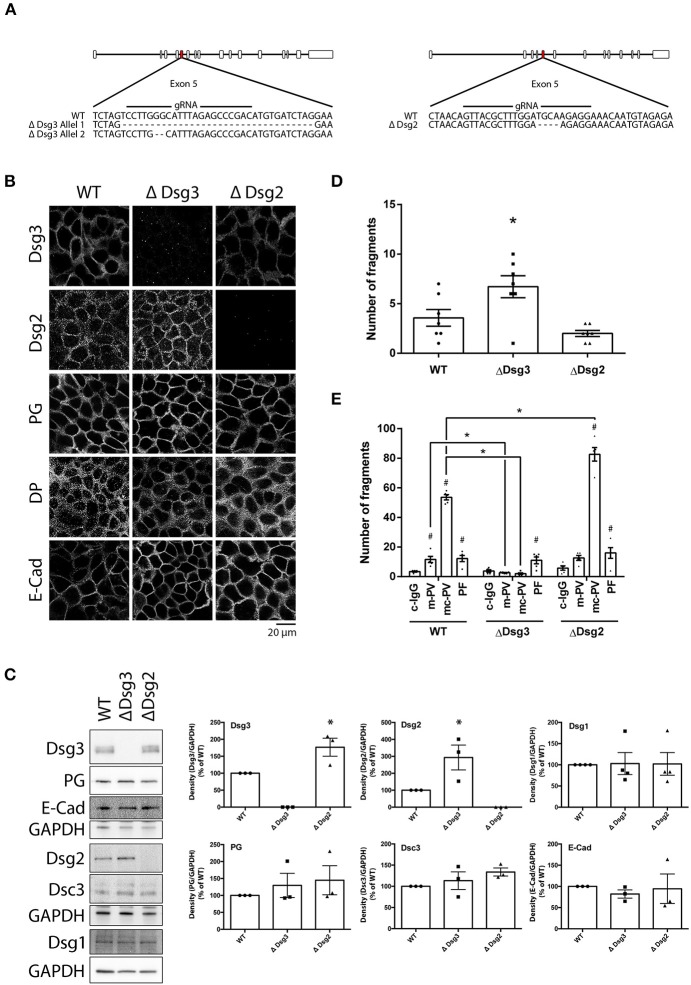
Genetic deletion of Dsg3 and Dsg2 using CRISPR/Cas9 in HaCaT keratinocytes. **(A)** Schematic of sequencing results after inducing a DSB with NHEJ repair in Exon 5 of either Dsg3 or Dsg2 using CRISPR/Cas9. **(B)** Cell line characterization by immunostaining of desmosomal proteins (*n* = 3). **(C)** Cell line characterization by immunoblot with representative images on the left and densitometric quantification on the right (*n* = 3, one-way ANOVA, *p ≤ 0.05 vs. WT). **(D)** Dispase assay of WT and Dsg-deficient cell lines under baseline treatment. (*n* = 7, one-way ANOVA, *p ≤ 0.05 vs. WT) **(E)** Cell lines incubated with IgG fractions for 24 h and subjected to Dispase assays (*n* = 5, two-way ANOVA, #*p* ≤ 0.05 vs. respective c-IgG, *p ≤ 0.05 vs. WT).

To delineate the role of Dsg3 for pemphigus autoantibody-induced signaling and loss of keratinocyte adhesion, HaCaT cell lines lacking either Dsg3 (ΔDsg3) or Dsg2 (ΔDsg2) were generated using the CRISPR/Cas9 gene editing technology. Generating Dsg1-deficient cells was not successful (unpublished observation). After expansion of monoclonal cultures, Sanger sequencing revealed one clone deficient for either Dsg2 or Dsg3. The Dsg3-deficient cell line presented a heterozygous deletion of 34 base pairs (bp) in one and 2 bp in a second allele in Exon 5. The cell line lacking Dsg2 presented a homozygous deletion of 4 bp in Exon 5. All genetic alterations resulted in a frameshift (Figure 5A). Deficiencies of Dsg2 or Dsg3 were validated by immunostaining and Western blot analyses (Figures 5B,C). Thereby, significant increase of Dsg2 was detectable in cells missing Dsg3 and Dsg3 was up-regulated in Dsg2-deficient keratinocytes. In contrast, protein levels and localization of plakoglobin (PG), desmoplakin (DP) or E-cadherin (E-Cad) remained unaltered in both lines. Interestingly, Dsg1 levels also remained unchanged in both cell lines. Next, Dsg-deficient cell lines were subjected to dispase-based dissociation assays. Baseline adhesion of keratinocytes lacking Dsg3 but not of cells missing Dsg2 was slightly reduced when compared to wild-type cells, which is in line with previous studies were both proteins were depleted by siRNA (30) (Figure 5D). Adhesion was still reduced after incubation of both PV-IgG fractions for 24 h in cells lacking Dsg2 but not in keratinocytes deficient for Dsg3, highlighting the importance of autoantibodies targeting Dsg3 for loss of cell adhesion in PV. PF-IgG on the other hand was still able to reduce adhesion significantly in Dsg3 deficient HaCaT cells (Figure 5E).

### 3.6. Dsg3-Dependence of Autoantibody-Induced Signaling

**Figure 6:**
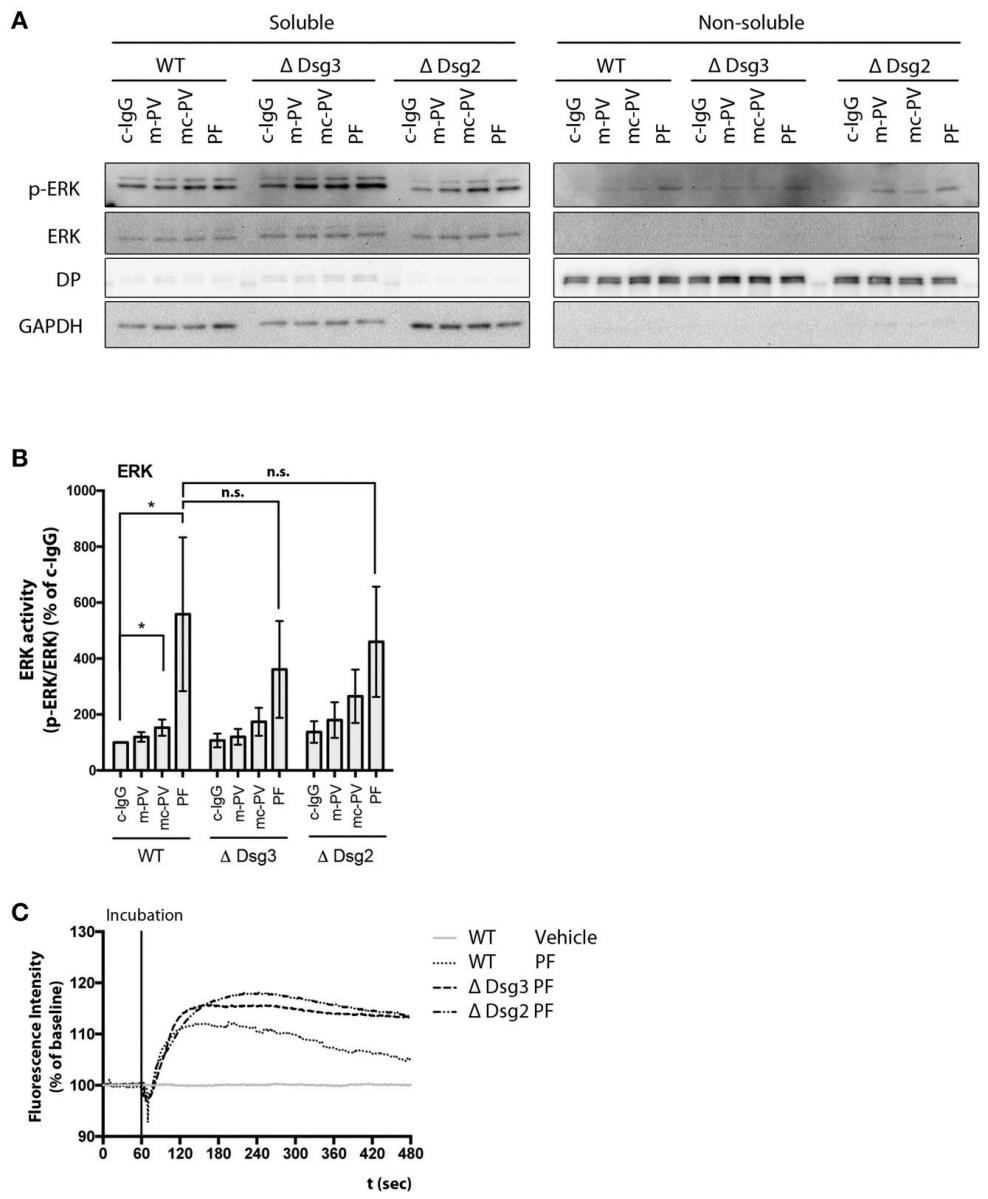
Signaling pathway modulation by pemphigus IgG fractions in Dsg-deficient cell lines. Analysis of ERK activation after 30 min application of IgG fractions by immunoblot in Dsg3- and Dsg2-deficient cell lines. **(A)** Representative immunoblot and **(B)** densitometric analysis (*n* = 9, two-way ANOVA, *p ≤ 0.05). **(C)** Representativ graph of Fura-2 ratiometric Ca^2+^ imaging reveals a PF-IgG mediated Ca^2+^ influx in all cell lines used (*n* = 3).

Lastly, ERK activity and Ca^2+^ influx were studied in keratinocytes deficient for either Dsg3 or Dsg2. Cell monolayers were incubated for 30 min with the respective IgG fractions and ERK phosphorylation was evaluated in immunoblots. Comparable to WT keratinocytes, PF-IgG significantly activated ERK in Dsg3- and Dsg2-deficient cells whereas PV-IgG-induced ERK phosphorylation was not consistent (Figures 6A,B). Additionally, PF-IgG mediated Ca^2+^ influx was evaluated by ratiometric Fura-2 imaging. A transient increase of intracellular Ca^2+^ was observed in all cell lines without major differences (Figure 6C). These results indicate that Dsg3 and Dsg2 are not required for activation of ERK and influx of Ca^2+^, at least in response to PF-IgG.

## 4. Discussion

**Figure 7:**
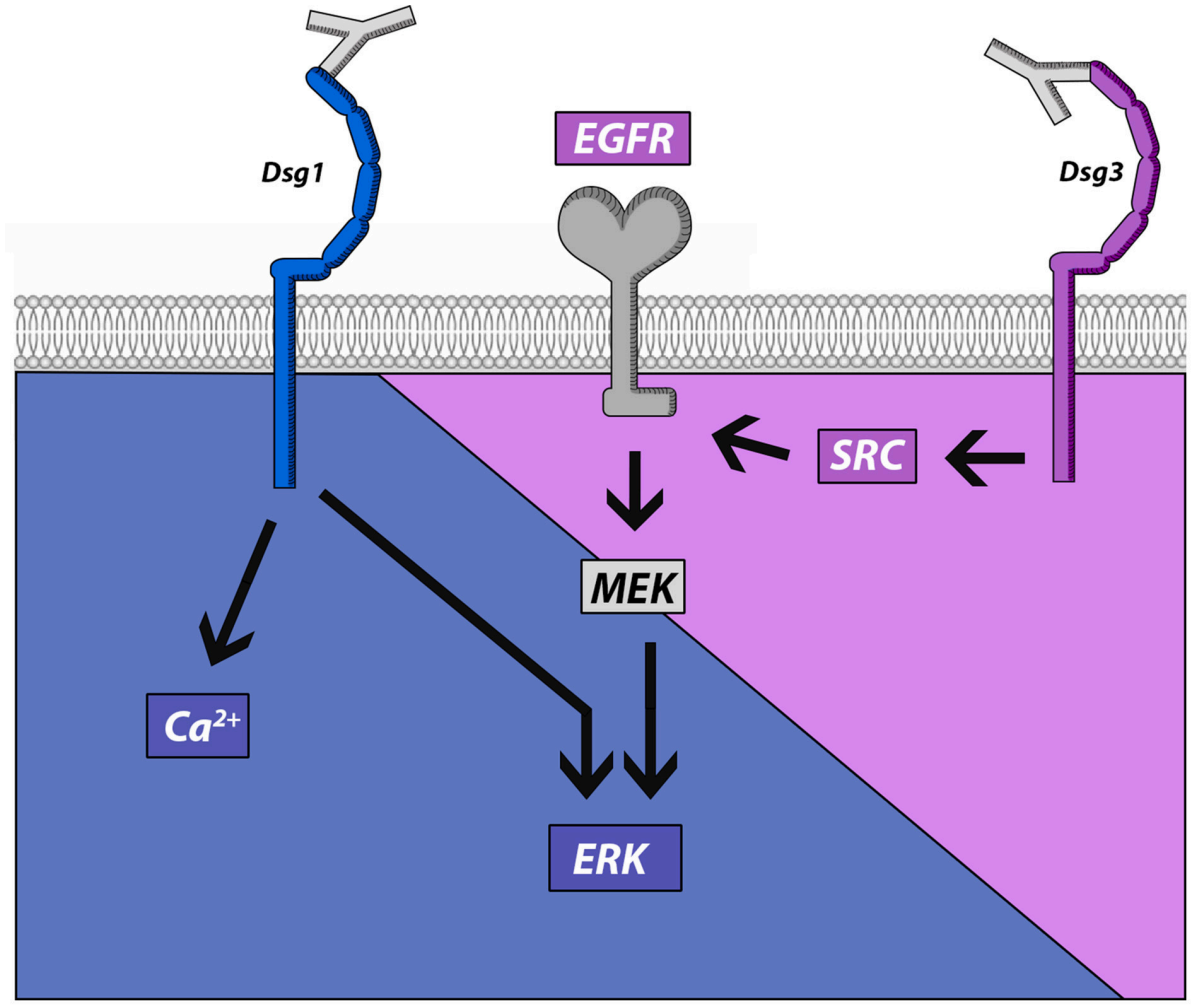
Schematic of signaling pathways activated by pemphigus autoantibodies directed against Dsg1 and Dsg3. Src and EGFR activation is associated with antibodies targeting Dsg3 whereas autoantibody fractions against Dsg1 induce ERK activation and Ca^2+^ influx.

This study demonstrates that IgG-induced signaling mechanisms in keratinocytes correlate with different autoantibody profiles in PV and PF. PV-IgG but not PF-IgG activated EGFR in a Src-dependent manner suggesting that Dsg1 is not required for EGFR activation. On the other hand, both PV-IgG and PF-IgG activated ERK and induced Ca^2+^ influx when antibodies targeting Dsg1 were present. In Dsg3-deficient keratinocytes, in which PV-IgG-induced loss of cell adhesion was abolished, ERK activation and Ca^2+^ influx were preserved in response to PF-IgG. These results indicate that these signaling pathways were indeed independent from Dsg3, but were in the absence of Dsg3, at least when induced by mc-PV, not able to reduce adhesion (Figure 7). Nevertheless, because inhibition of EGFR strongly reduced loss of cell adhesion in response to PV-IgG but not PF-IgG and Ca^2+^ chelation significantly reduced effects of both PV-IgG and PF-IgG, we conclude that both Dsg3- and Dsg1-dependent signaling mechanisms contribute to loss of keratinocyte adhesion in pemphigus.

### 4.1. Role of Signaling Pathways for Loss of Cell Adhesion in Pemphigus

It is generally accepted that loss of keratinocyte adhesion in pemphigus is mediated via both direct inhibition of Dsg3 binding and several signaling mechanisms (7). Amongst the plethora of signaling events implicated in pemphigus pathogenesis, p38MAPK, EGFR, Src and PKC have been suggested to be central for regulation of desmosome turn-over (5, 31). Indeed, inhibition of these pathways was protective in PV mouse models *in vivo* and human epidermis *ex vivo* (32–37). Activation of EGFR in response to PV-IgG was shown after 30-60 min *in vitro* (38, 39) and by AK23 after 120 min *in vivo* (23). This is in line with results shown here in which EGFR was significantly activated after 30 min. We found that EGFR activation in response to PV-IgG was blocked by Ca^2+^ chelation when compared to c-IgG, indicating that EGFR activation is dependent on Ca^2+^. Moreover, we demonstrated that PV-IgG-induced EGFR activation is Src-dependent, which is in line with a previous report (39). Similarly, EGFR activation in response to PV-IgG has been reported downstream of p38MAPK (40). Other studies showed that Src is downstream of EGFR signaling (41). This discrepancy may be explained by different mechanisms to activate EGFR. Similar to our study using PV-IgG, it was shown for AK23 in mice that EGFR was phosphorylated at Y845 after 2 h, which is typically a site for Src-mediated trans-activation (23). However, phosphorylation at the ligand-mediated activation site Y1173 was observed after 24 h as well. Since EGFR family ligands such as EGF and betacellulin are upregulated upon PV-IgG incubation (41), it is possible that both canonical ligand-mediated and more rapid Src-induced transactivation of EGFR occur. In a previous study, in contrast to EGF, loss of cell adhesion and Dsg3- and Dsg1-mediated binding in response to PV-IgG were not blocked by inhibitors of EGFR or Src (28). Moreover, EGFR phosphorylation at Y1173 and Y845 was induced by EGF but not by four different PV-IgG fractions. However, EGFR activation was tested after 60 min only and meanwhile efficacy of EGFR inhibitors to block autoantibody-induced skin blistering was reported to be strictly dose-dependent (34). Therefore, based on the latter study, a role of EGFR for PV-IgG-induced loss of keratinocyte adhesion cannot be ruled out. Nevertheless, these data suggest that not in all PV patients EGFR and Src contribute to skin blistering.

It is not entirely clear how EGFR regulates desmosomal adhesion. EGFR may reduce cell adhesion together with ADAMs via shedding of Dsg isoforms and inhibition of desmosome assembly as has been shown for Dsg2 (42, 43). In line with this, AFM revealed that EGF similar to PV-IgG reduced Dsg3 binding frequency. However, EGF- but not PV-IgG-induced loss of Dsg3 binding was Src-dependent. Since PV-IgG cause direct inhibition of Dsg3 binding on the molecular level, it is plausible that Src inhibition cannot override this effect as shown previously for p38MAPK (28, 29). Nevertheless, these data indicate that EGFR via Src can contribute to loss of Dsg3 adhesion similar as shown before for Dsg2 in enterocytes (27). Alternatively, EGFR may activate ERK, which is activated in response to PV-IgG and PF-IgG (15, 38), Here, we showed that ERK activation by mc-PV-IgG was significant after 30 min and was dependent on EGFR and Src. In contrast, PF-IgG-induced ERK activation was detected after 30 min and after 60 min but was independent of Src and MEK and not paralleled by EGFR activation. Nevertheless, EGFR inhibition blunted PF-IgG-induced ERK activation indicating that baseline EGF activity was required. Taken together, these data suggest that PF-IgG and mc-PV-IgG activated ERK by different mechanisms.

Besides the functional interplay of EGFR, Src and ERK, we further characterized the role of Ca^2+^ for autoantibody-induced loss of keratinocyte adhesion. As the first signaling mechanism demonstrated to be activated by pemphigus autoantibodies (24), Ca^2+^ influx was shown to be induced within 10-80 sec and shown to trigger PKC activation (25, 44). However, so far it remained unclear whether Ca^2+^ contributes to loss of adhesion. The data presented here, which show that Ca^2+^ chelation reduces loss of cell cohesion in response to all pemphigus autoantibodies, would be in line with this hypothesis. Moreover, mc-PV-IgG- but not PF-IgG-induced ERK activation was blocked by Ca^2+^ chelation, suggesting that for ERK activation Ca^2+^ influx is not absolutely required, at least when induced by PF-IgG.

### 4.2. Function of Dsg1 and Dsg3 to Trigger Signaling and Loss of Cell Cohesion in Pemphigus

In contrast to Dsg1, pemphigus autoantibodies against Dsg3 directly interfere with Dsg interaction (15, 18, 28), which alone is not sufficient to cause loss of keratinocyte adhesion (29). This suggests that signaling is important for loss of keratinocyte adhesion in pemphigus and regulation of signaling pathways downstream of autoantibodies may differ and might be responsible to induce different clinical phenotypes in pemphigus. Some signaling pathways such as p38MAPK, which has been shown to inhibit the small GTPase RhoA in keratinocytes, were found to be activated by both PV-IgG and PF-IgG and contribute to blistering and desmosome alterations in mouse and human epidermis (15, 32, 37, 45–47). In contrast, other signaling pathways correlate with autoantibody profiles against Dsg1 and Dsg3. We demonstrate that both Ca^2+^ influx and ERK activation were observed in the presence of autoantibodies targeting Dsg1 only (mc-PV-IgG and PF-IgG), which is in line with previous observations (15). In contrast, Src-dependent EGFR activation was found in response to PV-IgG containing antibodies against Dsg3. To clarify the role of Dsg3 in this context, we established Dsg3-deficient HaCaT cells by CRISPR/Cas9 mediated gene editing. We found that ERK activation and Ca^2+^ influx in response to PF-IgG were preserved in both cells lines whereas ERK activation induced by mc-PV-IgG was blunted. These data suggest that Ca^2+^ influx and ERK activation are mediated by antibodies not targeting Dsg3, which may include antibodies against Dsg1, whereas EGFR via Src is activated by antibodies not targeting Dsg1 which may comprise antibodies against Dsg3 (Figure 7). In line with this, in a previous study we found that AK23, which is specific for Dsg3, did not induce Ca^2+^ influx in human keratinocytes (15). Nevertheless, since siRNA-mediated depletion of Dsg1 and Dsg3 was not sufficient to significantly reduce PV-IgG-mediated EGFR and Src activation, it is possible that autoantibodies against other targets are involved in this process as well (39). These may include antibodies against Dsc3, M3 muscarinic acetylcholine receptor, Secretory Pathway Ca^2+^ ATPase (SPCA)1, Human Thyroid Peroxidase (TPO), Thyroglobulin (Tg) or others (11, 48). In this scenario, it is not entirely clear how PF-IgG induces ERK activation. ERK was activated independent of Src and MEK indicating that PF-IgG regulates ERK signaling by other mechanisms (Figure 7). It was shown that Dsg1 binds to Erbin to suppress EGFR-induced ERK activation via Erbin-mediated sequestration of SHOC2 to allow epidermal differentiation (49, 50). Therefore, it is possible that antibodies against Dsg1 may be required to disinhibit ERK activation which is in line with our observation that baseline EGFR activity is required for PF-IgG-induced ERK activation.

### 4.3. Antibodies Targeting Dsg3 Contribute to Loss of Cell Cohesion in Pemphigus

Dsg1 appears to be more critical for epidermal integrity compared to Dsg3 because Dsg3-deficient mice develop mild skin lesions, which spontaneously heal, whereas Dsg1-deficient mice completely lose superficial epidermal layers during birth and die within 24 h (51, 52). However, Dsg3-specific antibodies were effective to cause skin blistering in mice (53) whereas in patients as well as in human epidermis *ex vivo* antibodies against Dsg3 alone usually are not sufficient to cause skin blisters (1, 37). Nevertheless, in non-lesional skin of m-PV patients Dsg3 distribution was altered but on the ultrastructural level size and number of desmosomes remained normal, whereas in skin lesions and experiments using autoantibodies from mc-PV and PF patients desmosome size and keratin insertion were reduced as well (37, 54–56). These observations indicate that autoantibodies against Dsg3 may contribute to loss of keratinocyte adhesion. In line with this, it was demonstrated recently that immune-adsorption of autoantibodies targeting Dsg3 and Dsg1 abolished PV-IgG-induced blistering whereas Dsg3-specific antibodies alone were sufficient to induce blisters in mice (57). Therefore, it is important to characterize the role and mechanisms of autoantibodies targeting Dsg3 in pemphigus. We observed that Ca^2+^ influx and ERK activation were preserved in Dsg3-deficient cells but not sufficient to cause md-PV-IgG mediated loss of keratinocyte cohesion. Since Dsg2 was up-regulated in Dsg3-deficient cells we cannot rule out completely that enhanced expression contributed to out-balance pathogenic effects of autoantibodies. Nevertheless, we conclude that autoantibodies in m-PV, which include antibodies targeting Dsg3, may interfere with desmosome turn-over via direct inhibition of Dsg3 binding and several signaling pathways such as Src, EGFR and p38MAPK and thereby sensitize desmosomes for autoantibodies against Dsg1 and other antigens which together induce an mc-PV phenotype. This would also provide further explanation to why epidermal blistering in PV occurs in supra-basal rather than in the superficial epidermis as in patients suffering from PF.

## Data Availability

The raw data supporting the conclusions of this manuscript will be made available by the authors, without undue reservation, to any qualified researcher.

## Ethics Statement

Patients gave written informed consent for research use of their blood sera. It was additionally approved by the Ethics Committee of the University of Marburg Medical Faculty.

## Author Contributions

EW, M-TW, and MS performed and analyzed experiments. FV performed and analyzed AFM data. RP and RE provided patient sera. EW and JW analyzed and interpretated the data, designed the study and wrote the manuscript.

### Conflict of Interest Statement

The authors declare that the research was conducted in the absence of any commercial or financial relationships that could be construed as a potential conflict of interest.
